# Favorable control of hepatocellular carcinoma with peritoneal dissemination by surgical resection using indocyanine green fluorescence imaging: a case report and review of the literature 

**DOI:** 10.1186/s13256-022-03440-5

**Published:** 2022-06-06

**Authors:** Yuma Tani, Hiroki Sato, Ryuichi Yoshida, Kazuya Yasui, Yuzo Umeda, Kazuhiro Yoshida, Tomokazu Fuji, Kenjiro Kumano, Kosei Takagi, Masaaki Kagoura, Takahito Yagi, Toshiyoshi Fujiwara

**Affiliations:** Department of Gastroenterological Surgery, Okayama University Hospital, 2-5-1 Shikata-cho, Kita-ku, Okayama, 700-8558 Japan

**Keywords:** Hepatocellular carcinoma, Peritoneal dissemination, Indocyanine green fluorescence

## Abstract

**Background:**

The optimal management for peritoneal dissemination in patients with hepatocellular carcinoma remains unclear. Although several reports have described the usefulness of surgical resection, the indications should be carefully considered. Herein, we report the case of a patient with hepatocellular carcinoma with peritoneal recurrence who underwent surgical resection using an indocyanine green fluorescence navigation system and achieved favorable disease control.

**Case presentation:**

A 45-year-old Asian woman underwent left hemihepatectomy for a ruptured hepatocellular carcinoma. Seventeen months after the initial surgery, a single nodule near the cut surface of the liver was detected on computed tomography, along with elevation of tumor markers. The patient was diagnosed with peritoneal metastasis and underwent a surgical resection. Twelve months later, a single nodule on the dorsal side of the right hepatic lobe was detected on computed tomography, and we performed surgical resection. Indocyanine green (0.5 mg/kg) was intravenously administered 3 days before surgery, and the indocyanine green fluorescence imaging system revealed clear green fluorescence in the tumor, which helped us perform complete resection. Indocyanine green fluorescence enabled the detection of additional lesions that could not be identified by preoperative imaging, especially in the second metastasectomy. There was no further recurrence at 3 months postoperatively.

**Conclusion:**

When considering surgical intervention for peritoneal recurrence in patients with hepatocellular carcinoma, complete resection is mandatory. Given that disseminated nodules are sometimes too small to be detected by preoperative imaging studies, intraoperative indocyanine green fluorescence may be an essential tool for determining the indications for surgical resection.

## Background

Peritoneal dissemination is a rare form of recurrence in hepatocellular carcinoma (HCC), and it has been reported that implantation of tumor cells by rupture of the primary tumor [1], needle biopsy, and radiofrequency ablation (RFA) [2] can cause peritoneal dissemination. Patients with HCC with peritoneal recurrence have poor prognosis, but surgery has been reported to improve prognosis [3]. Indocyanine green (ICG), which fluoresces green when irradiated with near-infrared light, has been used not only for assessment of liver function but also for surgical navigation. Recent reports have shown that the ICG fluorescence pattern is useful for distinguishing between tumor and normal tissues for both primary and extrahepatic lesions of HCC [4–6]. It has also been reported that ICG accumulates better in highly differentiated than moderately or poorly differentiated HCC [7]. In this report, we describe the case of a patient with HCC with peritoneal recurrence whose disease was well controlled by a surgical approach, including the ICG fluorescence navigation system. We also performed surgical resection twice for peritoneal dissemination of HCC using indocyanine green fluorescence imaging, including microlesions that could not be identified preoperatively.

## Case presentation

**Fig. 1:**
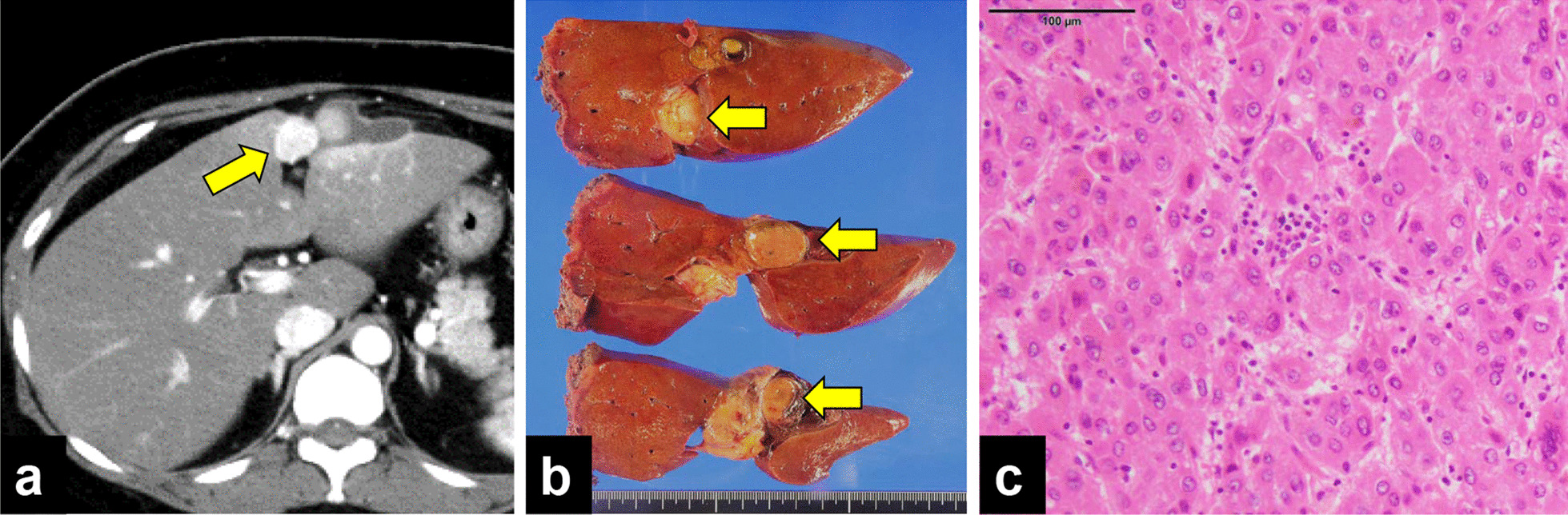
Findings of initial liver resection. **a** Contrast-enhanced abdominal CT images showing a 45-mm-diameter tumor located in segment 3/4. The arrow points to the tumor site. **b** Cut surface of the tumor. Ruptured tumor is observed. The arrows points to the tumor. **c** An image of the lesion stained with hematoxylin and eosin; the tumor was confirmed as moderately differentiated hepatocellular carcinoma

A 45-year-old Asian woman with history of diabetes mellitus visited her family doctor with a chief complaint of epigastric pain. Contrast computed tomography (CT) revealed a 45-mm-diameter tumor with extravasation in liver segment S3/4, indicating HCC rupture (Fig. 1a). Transcatheter arterial embolization (TAE) was performed immediately, and the patient was referred to our hospital for radical surgery. The serum tumor markers alpha-fetoprotein (AFP) and protein induced by vitamin K absence or antagonist-II (PIVKA-II) were markedly elevated (AFP: 3367 ng/mL, PIVKA-II: 885 mAU/mL). She was negative for hepatitis B and C, but had a fatty liver. Liver function was normal (Child–Pugh score, 5 points; grade A). We performed a left hemihepatectomy (Fig. 1b) 1 month after TAE. Histologically, the tumor was confirmed to be moderately differentiated HCC (Fig. 1c). There was no vascular invasion, lymph node metastasis, or intrahepatic metastasis. The surgical margins were negative. The pathological stage was II, according to the staging system of the Liver Cancer Study Group of Japan [8].

**Fig. 2:**
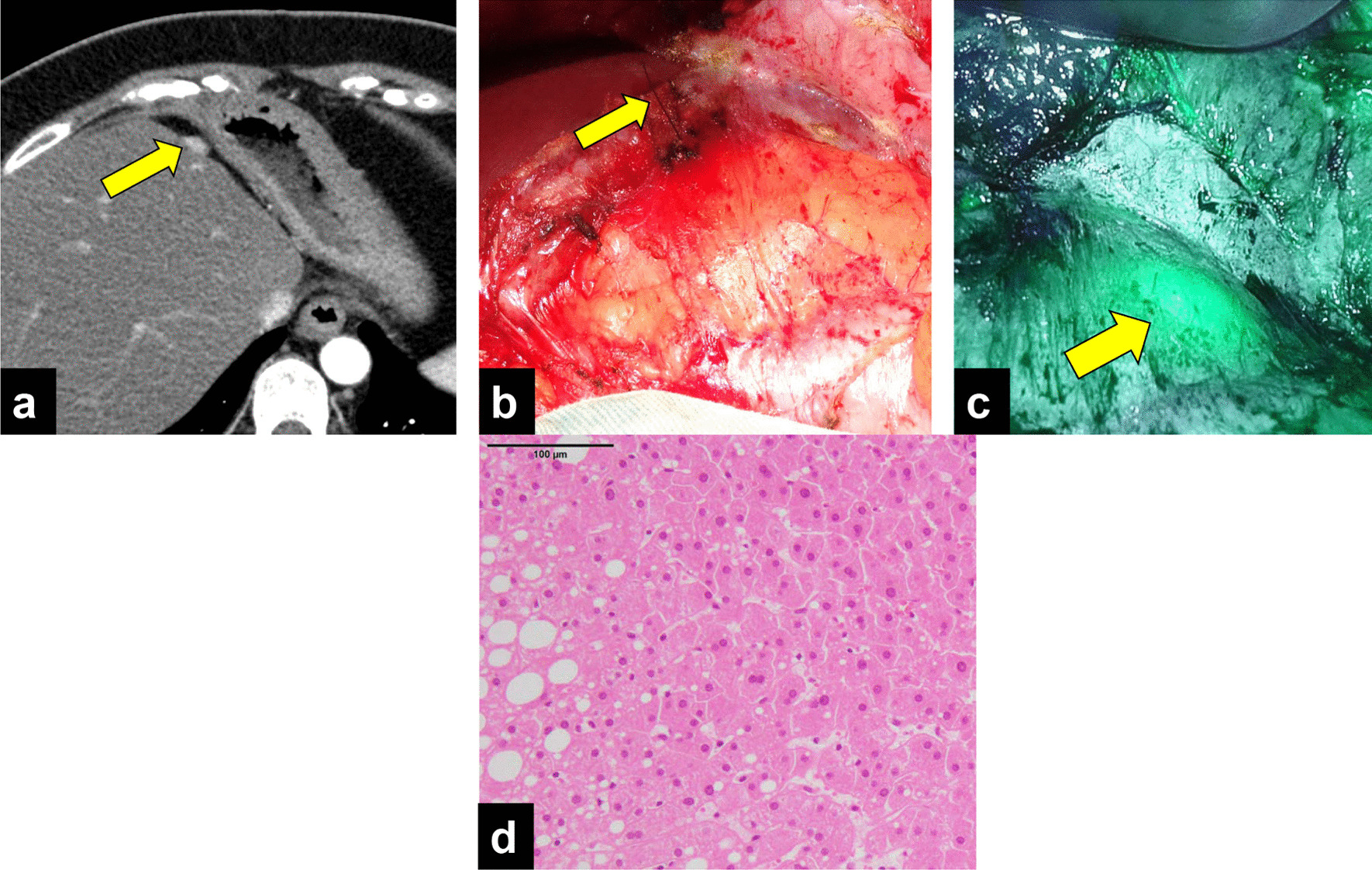
Findings of first resection of peritoneal dissemination. **a** Preoperative image showing a 10-mm-diameter lesion near the cut surface of the liver. The arrow points to the tumor site. **b** The arrow points to the string of a metallic marker, but the location of tumor was unclear. **c** ICG fluorescence imaging revealed green fluorescence clearly at the tumor site. The arrow points to the tumor site. **d** HE image. Histology of peritoneal dissemination was similar to that of the specimen from the initial hepatectomy

Seventeen months after the initial surgery, CT revealed a 10-mm nodule near the cut surface of the liver (Fig. 2a), together with mild elevation of AFP and PIVKA-II (AFP: 76 ng/mL, PIVKA-II: 41 mAU/mL). We diagnosed peritoneal recurrence of HCC and performed laparotomy. Given that the tumor was undetectable by ultrasonography, ICG 0.5 mg/kg was administered intravenously 3 days before the surgery and a metallic marker was placed the day before the surgery for reliable identification and complete resection of the tumor. Intraoperative findings showed a metallic marker in the fatty tissue of the lesser omentum; however, the boundary of the tumor was unclear (Fig. 2b). Near-infrared imaging showed ICG accumulation with a clear boundary around the metallic marker and absence of other lesions, which enabled us to secure the surgical margin (Fig. 2c). Histopathological examination revealed enriched growth of atypical cells, indicating peritoneal dissemination of the HCC (Fig. 2d).

**Fig. 3:**
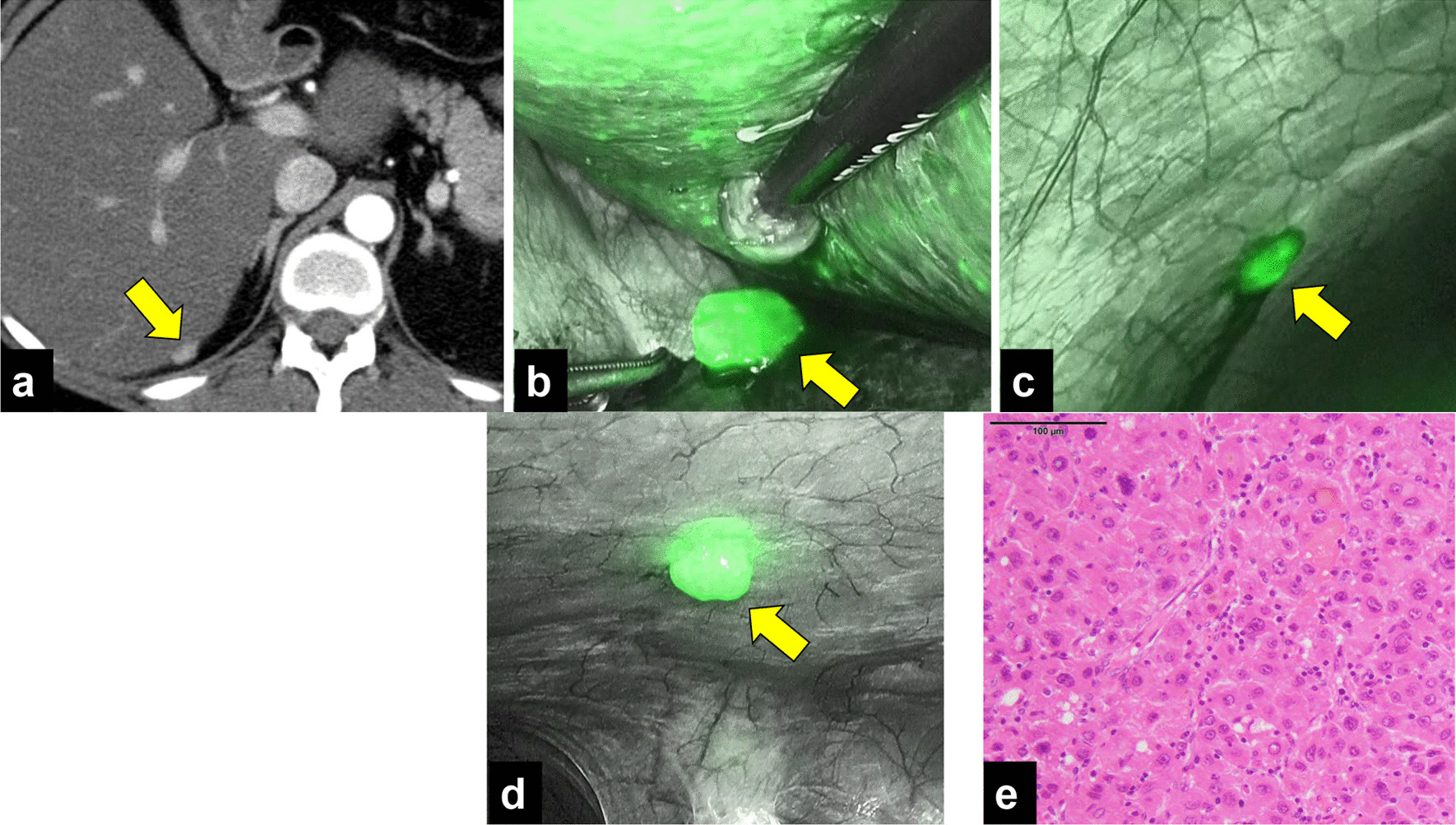
Findings of second resection of peritoneal dissemination. **a** Preoperative image showing a 12-mm-diameter lesion on the dorsal surface of the lower right lobe of the liver. No other lesions were detected on CT scan. **b** ICG fluorescence imaging revealed green fluorescence clearly at the tumor site. **c**, **d** Two small lesions, which were not identified preoperatively, were detected on the right diaphragm. ICG accumulation consistent with lesion. **e** HE image. All three lesions were diagnosed as recurrence of HCC. **a**–**d** The arrow points to the tumor site

Twelve months after resection of the peritoneal dissemination, a CT scan revealed a 12-mm nodule on the dorsal side of the lower part of the right liver lobe (Fig. 3a) with elevation of AFP and PIVKA-II (AFP: 72 ng/mL, PIVKA-II: 47 mAU/mL). Preoperative imaging analysis showed no other lesions; therefore, we decided to repeat surgical resection. Based on tumor location, we chose the laparoscopic approach, and ICG fluorescence imaging was used to identify the tumor (Fig. 3b). Intraoperatively, two disseminated lesions were detected by careful examination with ICG fluorescence (Fig. 3c, d). All lesions were resected, and R0 resection was achieved, resulting in immediate normalization of tumor markers. Histopathologically, all the lesions were diagnosed as peritoneal dissemination of HCC (Fig. 3e). The patient is alive and monitored in an outpatient setting; no recurrence has been noted 3 months after the second metastasectomy.

## Discussion and conclusions

Peritoneal recurrence is a rare form of HCC. According to the report of the 22nd Nationwide Follow-Up Survey of Primary Liver Cancer in Japan (2012–2013) [8], of 7494 resected HCC cases, only 28 (0.4%) had peritoneal dissemination. Implantation of tumor cells at time of needle biopsy, RFA, surgical resection, and tumor rupture is the main cause of peritoneal dissemination [1]. There is no clear evidence regarding the treatment of peritoneal HCC recurrence. While peritoneal dissemination in other types of cancer is usually widespread and not indicated for surgery, peritoneal dissemination in HCC is sometimes localized, and several reports have shown that surgical resection of peritoneal recurrence can improve the prognosis of patients with HCC [11, 12]. In our case, the tumor cells were speculated to have been implanted by the rupture of the primary tumor. The disease was well controlled by surgical resection of the recurrent lesion, and the patient is alive 33 months after the initial surgery. Oligoperitoneal recurrence in patients with HCC may receive some benefit from surgical resection.

ICG is used not only for evaluation of liver function but also for surgical navigation, such as detection of tumors or blood flow assessment. ICG fluorescence can be used to detect both primary and extrahepatic lesions in HCC. Sato *et al.* [13] reported that the positive predictive value of ICG fluorescence by near-infrared observation in 33 extrahepatic metastatic lesions was 100%, whereas the negative predictive value was 86%. In our case, intraoperative ICG fluorescence imaging was useful for the resection of peritoneal dissemination for the following reasons: First, we were able to clearly distinguish the tumor from the surrounding normal tissue. Especially for the first metastasectomy, the tumor was deeply embedded in the fatty tissue and it was difficult to macroscopically recognize the boundary of the tumor. Second, small lesions that could not be identified by preoperative CT could be detected by ICG fluorescence imaging, and complete resection was achieved. In particular, laparoscopic surgery allows observation of the entire abdominal cavity in a minimally invasive manner. Considering that R0 resection is one of the minimum requirements for surgical resection for peritoneal recurrence, a laparoscopic approach in combination with ICG fluorescence can be a promising strategy to determine the surgical indication.

To assess the efficacy of ICG fluorescence for resection of peritoneal dissemination in patients with HCC, we conducted a literature search using the PubMed database and found seven cases of surgical resection for peritoneal dissemination of HCC using ICG fluorescence imaging, including our case [4–6, 10, 14] (Table 1). There were two cases of rupture of primary HCC (cases 2 and 7), and TAE was performed in all cases. In case 4, ICG fluorescence imaging at the time of the initial surgery revealed that the patient had peritoneal metastasis in the omentum. Consequently, surgery for primary HCC was cancelled. In five of the seven cases, the authors were able to intraoperatively identify the lesions using ICG fluorescence that could not be detected by preoperative image analysis. Laparoscopic surgery was performed in three cases, and in two of the three cases, new lesions were detected at a site distant from the main lesion. Regarding the prognosis after resection of peritoneal dissemination, recurrence was observed in case 5 and in our case. In case 5, eight lesions were resected, including those identified intraoperatively by ICG fluorescence, with a maximum diameter of 28 mm, and peritoneal re-recurrence was observed only 2 months after the surgery. Our case also showed multiple lesions with peritoneal recurrence; therefore, careful follow-up of the patient is necessary. Given that R0 resection is the key to surgical resection of peritoneal dissemination, careful laparoscopic examination of the abdominal cavity using ICG fluorescence is an important step and may allow the selection of patients who will truly benefit from surgical resection of peritoneal dissemination. Indeed, in case 4, ICG fluorescence imaging helped physicians decide on the treatment strategy. Systemic treatment, rather than surgical resection, was selected. In our case, surgery was performed 3 days after ICG administration. For hepatic tumors, it is recommended to administer ICG at least 2 days before surgery for clear contrast between tumors and background liver tissue [7]; however, for extrahepatic metastases, there is no need to consider contrast with the liver tissue, so 1–5 days between ICG administration and surgery may be sufficient [13].

**Table 1:** Reported cases of surgical resection for peritoneal dissemination of HCC detected by ICG fluorescence imaging

Case	Study	Age/sex	Rupture of primary tumor	Former treatment	Surgical approach	ICG timing	ICG amount (mg/kg)	No. of metastases	Recurrence site	Size of maximum tumor (mm)	Newly detected tumors	Recurrence after resection of peritoneal dissemination
1	Nakamura *et al.*	76/M	N	HTX + RFA + TACE	Open	2 days prior	0.5	2	Abdominal wall, omentum	ND	Y	N
2	Miyazaki *et al.*	75/M	Y	TAE + HTX	Lap	3 days prior	0.5	1	Retroperitoneum	13	N	N
3	He *et al.*	46/M	N	HTX	Open	ND	ND	1	Peritoneum of right lower abdomen	90	N	N
4	He *et al.*	77/M	N	N	Open	ND	ND	1	Omentum	ND	Y	N (enlargement of concurrent liver tumor)
5	Hayashi *et al.*	57/M	N	HTX	Open	2 days prior	0.5	8	Peritoneum of abdominal wall (right upper, right flank, epigastrium, central)	28	Y	Y (peritoneal dissemination)
6	Sasaki *et al.*	76/M	N	HTX + RFA + TACE	Lap	1 day prior	0.5	3	Retroperitoneum, right diaphragm	10	Y	N
7-1	Our case (first resection)	46/F	Y	TAE + HTX	Open	3 days prior	0.5	1	Omentum	17	N	Y (peritoneal dissemination)
7-2	Our case (second resection)	47/F	Y	TAE + HTX + resection of peritoneal dissemination	Lap	3 days prior	0.5	3	Retroperitoneum, right diaphragm	12	Y	N

*HTX* hepatectomy, *RFA* radiofrequency ablation, *TACE* transarterial chemoembolization, *Lap* laparoscopic surgery

In conclusion, we report the case of a patient with HCC who underwent surgical resection for peritoneal dissemination. ICG fluorescence imaging may be useful for determining the indications for surgical resection of peritoneal dissemination.

## Data Availability

Not applicable.

## Abbreviations

HCC: Hepatocellular carcinoma
ICG: Indocyanine green
RFA: Radiofrequency ablation
CT: Computed tomography
TAE: Transcatheter arterial embolization
AFP: Alpha-fetoprotein
PIVKA-II: Protein induced by vitamin K absence or antagonist II
